# Complete Genome of *Bacillus pumilus* Siphophage Glittering

**DOI:** 10.1128/genomeA.00856-13

**Published:** 2013-12-05

**Authors:** Solomon P. Matthew, Skyelar L. Decker, Karthik R. Chamakura, Gabriel F. Kuty Everett

**Affiliations:** Center for Phage Technology, Texas A&M University, College Station, Texas, USAa; Department of Integrated Science and Technology, James Madison University, Harrisonburg, Virginia, USAb

## Abstract

*Bacillus pumilus* is a Gram-positive bacterium widely used in agriculture both as an antifungal and as a growth-promoting symbiont. *B. pumilus* is rarely infectious but has recently been shown to infect humans. Here, we present the complete genome of *B. pumilus* phage Glittering, a potential biocontrol agent for *B. pumilus*.

## GENOME ANNOUNCEMENT

*Bacillus pumilus* is a Gram-positive spore-forming bacterium. It resides in the soil and colonizes the root area of plants. *B. pumilus* is used in agriculture for its growth-stimulating and antifungal/antibacterial activities (1–3). Cases of human infection by *B. pumilus* are rare, but in 2006, 3 cases of food poisoning from contaminated rice were reported (4). In 2007, 3 case studies were published concluding that a strain of *B. pumilus* was responsible for the development of cutaneous lesions morphologically similar to those caused by *B. anthracis* (5). The use of bacteriophages for controlling pathogens is gaining attention as the occurrence of antibiotic resistance is on the rise. Here, we report the complete genome of *B. pumilus* phage Glittering.

*B. pumilus* strain BL-8 was isolated on the campus of James Madison University (6). Phage Glittering was obtained from a soil sample collected in Harrisonburg, VA. Phage DNA was sequenced using 454 pyrosequencing at the Emory GRA Genome Center (Emory University, Atlanta, GA). The trimmed FLX Titanium reads were assembled to a single contig at 299.6-fold coverage using the Newbler assembler version 2.5.3 (454 Life Sciences) with the default settings. PCR confirmed the completed contigs. Genes were predicted using GeneMarkS (7), and gene predictions were corrected using software tools available on the Center for Phage Technology (CPT) portal (https://cpt.tamu.edu/cpt-software/portal/). Transmission electron microscopy was performed at the University of Mary Washington.

The unit genome of Glittering has 48,395 bp, with a coding density of 91.9% and a G+C content of 42%. Seventy-seven unique coding sequences were identified, of which 51 encode hypothetical conserved or novel proteins and 26 have a predicted function based on BLASTp and InterPro analysis (8, 9).

Encoded proteins related to DNA replication and recombination include DNA helicase, primase, DNA polymerase III subunits alpha and epsilon, Holliday junction resolvase, and two nucleases. The identified genes for phage morphogenesis proteins encode a minor head protein, scaffold protein, major capsid protein, tail completion protein, tape measure protein, and a tailspike protein with a pectin lyase domain. Tailspike proteins containing pectin lyase domains have been reported to depolymerize exopolysaccharide (EPS) (10). A TerL protein was found that has homology to the TerLs of phages with long terminal repeats. An examination of the raw sequencing data using the Pause (https://cpt.tamu.edu/cpt-software/releases/pause/) method and comparison to a similar phage (Riggi; GenBank accession no. KF669659) showed the terminal repeat to be 851 bp in length. Genes encoding DNA biosynthesis proteins (thymidylate synthase and deoxynucleotide monophosphate kinase) were also found. For lysis, Glittering uses a canonical holin/endolysin system consisting of a class-II holin (two transmembrane domains in an N-in C-in topology) and an *N*-acetylmuramoyl-l-alanine amidase.

An interesting find was an FtsK/SpoIIIE homolog. Gram-positive bacteria use SpoIIIE, an ATP-dependent DNA translocase, to pump chromosomal DNA across the forespore septum during sporulation. FtsK and SpoIIIE also remove proteins from the DNA during translocation (11, 12). The role of this protein during phage infection remains to be determined.

### Nucleotide sequence accession number.

The genome sequence of phage Glittering was contributed as accession no. KF669651 to GenBank.
